# Utility of the Neuropsychiatric Inventory Questionnaire (NPI-Q) in the assessment of a sample of patients with Alzheimer's disease in Chile

**DOI:** 10.1590/1980-57642016dn11-020005

**Published:** 2017

**Authors:** Gada Musa, Fernando Henríquez, Carlos Muñoz-Neira, Carolina Delgado, Patricia Lillo, Andrea Slachevsky

**Affiliations:** 1 Unidad de Neurología Cognitiva y Demencias, Hospital del Salvador, Providencia, Santiago, Chile; 2 Centro de Salud y Rehabilitación Capredena, La Florida, Santiago, Chile; 3 Departamento de Neurología y Neurocirugía, Hospital Clínico Universidad de Chile; 4 Centro de Investigación Avanzada en Educación. Universidad de Chile; 5 Departamento de Fisiopatología, ICBM y Departamento de Neurociencia Oriente, Facultad de Medicina, Universidad de Chile, Providencia, Santiago, Chile; 6 Centro de Gerociencias, Salud Mental y Metabolismo, Providencia, Santiago, Chile; 7 Servicio de Neurología, Clínica Alemana; 8 Universidad de los Andes, Santiago, Chile; 9 Departamento de Neurología Sur, Facultad de Medicina, Universidad de Chile

**Keywords:** dementia, assessment, neuropsychiatric inventory, Alzheimer's disease, prevalence, neuropsychiatric symptoms, demência, avaliação, inventário neuropsiquiátrico, doença de Alzheimer, prevalência, sintomas neuropsiquiátricos

## Abstract

The Neuropsychiatric Inventory Questionnaire (NPI-Q) is an informant-based instrument that measures the presence and severity of 12 Neuropsychiatric Symptoms (NPS) in patients with dementia, as well as informant distress.

**Objective:**

To measure the psychometric properties of the NPI-Q and the prevalence of NPS in patients with Alzheimer's disease (AD) in Chile.

**Methods:**

53 patients with AD were assessed. Subjects were divided into two different groups: mild AD (n=26) and moderate AD (n=27). Convergent validity was estimated by correlating the outcomes of the NPI-Q with Neuropsychiatric Inventory (NPI) scores and with a global cognitive efficiency test (Addenbrooke's Cognitive Examination - Revised - ACE-R). Reliability of the NPI-Q was analysed by calculating its internal consistency. Prevalence of NPS was estimated with both the NPI and NPI-Q.

**Results:**

Positive and significant correlations were observed between the NPI-Q, the NPI, and the ACE-R (r=0.730; p<0.01 and 0.315; p<0.05 respectively). The instrument displayed an adequate level of reliability (Cronbach's alpha=0.783). The most prevalent NPS were apathy/indifference (62.3%) and dysphoria/depression (58.5%).

**Conclusion:**

The NPI-Q exhibited acceptable validity and reliability indicators for patients with AD in Chile, indicating that it is a suitable instrument for the routine assessment of NPS in clinical practice.

## Abstract (pt)

O Questionário de Inventário Neuropsiquiátrico (NPI-Q) é um instrumento baseado em informantes que mede a presença e a gravidade de 12 Sintomas Neuropsiquiátricos (NPS) em pacientes com demência, bem como o sofrimento do informante.

**Objetivo:**

Avaliar as propriedades psicométricas do NPI-Q e a prevalência de NPS em pacientes com doença de Alzheimer (DA).

**Métodos:**

Foram avaliados 53 pacientes com DA. Eles foram divididos em dois grupos diferentes: AD leve (n=26) e AD moderado (n=27). A validade convergente foi estimada correlacionando os resultados do NPI-Q com os escores do Inventário Neuropsiquiátrico (NPI) e um teste de eficiência cognitiva global (Addenbrooke's Cognitive Examination - Revised - ACE-R). A confiabilidade do NPI-Q foi analisada pelo cálculo da sua consistência interna. A prevalência de NPS foi estimada com NPI e NPI-Q.

**Resultados:**

Foram observadas correlações positivas e significativas entre NPI-Q, NPI e ACE-R (r=0,730; p<0,01 e 0>315; p<0>05). O instrumento apresentou um nível adequado de confiabilidade (alfa de Cronbach=0J83). Os NPS mais prevalentes foram apatia/indiferença (62,3%) e disforia/depressão (58,5%).

**Conclusão:**

O NPI-Q apresenta indicadores de validade e confiabilidade aceitáveis em pacientes com DA, o que indica que é um instrumento adequado para a avaliação rotineira de NPS na prática clínica.

## INTRODUCTION

Life expectancy in Latin America has rapidly increased over recent years. Chile has one of the highest rates of population ageing within the zone, with 11.4% growth in individuals aged 60 or older.^1,2^ Dementia is the most frequent mental disorder in this population and cases worldwide are expected to increase 68% by 2050. The estimated number of people with dementia in Chile was 150,293 in 2010, 181,761 in 2015 and is set to reach 533,188 in 2050.^3^ Additionally, the dependency rate associated with dementia is estimated to be roughly 37.8% higher than that of the general population. Concerning different types of dementia and their frequency, Alzheimer's disease (AD) is the most prevalent subtype, accounting for approximately 70% of all cases. The second most common cause of dementia is vascular dementia, accounting for 10-20% of cases, followed by Frontotemporal and Lewy Body dementias.^4,5^

Regardless of the type of dementia, besides its characteristic progressive cognitive and functional impairment, this syndrome is usually accompanied by behavioral disorders, also referred to as Neuropsychiatric Symptoms (NPS), which can be defined as non-cognitive/behavioral symptoms that include mood, perception, and behavior alterations.^6^ In terms of the likely emergence of NPS, several studies have indicated that more than 80% of patients with dementia experience at least one NPS,^6^ with increasing severity as the disease progresses.^7,8^ Similarly, data from the Cache County Study showed a 97% prevalence of at least one NPS with the most frequent symptoms being depression (77%), apathy (71%) and anxiety (62%).^9^

There are different types of NPS, probably due to the various etiological mechanisms that underpin each type of dementia.^8-10^ Exploring the likely presence of NPS in patients with dementia is critical for an appropriate differential diagnosis. Their assessment is important given the relationship existing between the presence of NPS and decline in quality of life, increased institutionalization, and rise in patient mortality.^11^ These issues result in a high level of caregiver burden and greater economic costs because of the frequent use of medication.^9-12^

Complementary, NPS assessment is relevant in dementia care, considering that it might provide an insight into the management of the behavioural disorders emerging from the difficulties of each case, given that they become more frequent as the disease progresses.

In this context, appropriate assessment of NPS becomes extremely important for the differential diagnosis of dementia, its related clinical practice, and the assessment of patients' behavioural response to treatments.

One of the most common scales for assessing the presence and severity of NPS in dementia is the Neuropsychiatric Inventory (NPI),^13^ a carer-based tool that assesses the possible presence of 12 symptoms in dementia cases, including delusions, hallucinations, agitation/aggression, dysphoria/depression, anxiety, euphoria/elation, apathy/indifference, disinhibition, irritability/lability, aberrant motor behaviors, night-time behavioral disturbances and appetite/eating disturbances. This scale is very useful and widely employed in research; however, its administration is time-consuming for health professionals, discouraging its inclusion in everyday clinical practice. In order to tackle this drawback, Kaufer et al.^14^ developed a shorter version of the instrument: the Neuropsychiatric Inventory Questionnaire (NPI-Q), another informant-based instrument that is easier to administer in both clinical and research settings. This questionnaire assesses the possible presence of the same 12 NPS included in the NPI and also provides an index to grade the corresponding severity of each symptom and the respective distress it causes on the caregiver. The Spanish version of the NPI-Q developed by Boada et al. has shown appropriate validity and reliability in Spain.^15^ Nevertheless, in Latin America it has been validated only for Brazil^16^ and Mexico^17^ so far, while validation in other Latin American countries, including Chile, remains pending.

The main objective of this study was to assess the psychometric properties of the NPI-Q in Chile. The secondary objective was to compare the prevalence and severity of NPS in a Chilean sample comprising patients with AD.

## METHODS

**Participants.** A convenience sample of 53 patients with mild and moderate AD and their respective caregivers was included in this study. AD diagnoses were established by a neurologist based both on the Clinical Dementia Rating Scale (CDR)^18^ and on the National Institute of Neurological and Communicative Disorders and Strokes and the Alzheimer's Disease and Related Disorders Association - NINCDS-ADRDA - criteria.^19^ The subdivision of the sample into two different groups was done according to CDR Global Score: mild AD (n=26; CDR=1) and moderate AD (n=27; CDR>2). The inclusion criteria considered participants of both sexes, over 65 years old, Spanish speakers, and with at least 4 years of education. Patients were recruited from the Cognitive Neurology and Dementia Unit of the Neurology Service at the Hospital del Salvador and from the Neurology and Neurosurgery Service at the Hospital Clínico Universidad de Chile (HCUCH) in Santiago, Chile. Each participant was asked to sign an informed consent form, revised by the Ethics Committee of the West Metropolitan Health Service and that of the HCUCH in Santiago, Chile. All the subjects with AD underwent a full neurological examination, a neuropsychological assessment, and neuroimaging procedures (Structural Magnetic Resonance) to confirm their diagnoses. The exclusion criteria were severe sensory deficits (hearing and vision), a history of strokes, and major psychiatric disorders.

### Instruments

*Neuropsychiatric Inventory (NPI).* The NPI^13^ is a retrospective (1 month) caregiver/informant-based interview covering 12 NPS, including delusions, hallucinations, agitation/aggression, dysphoria/depression, anxiety, euphoria/elation, apathy/indifference, disinhibition, irritability/lability, aberrant motor behaviors, night-time behavioral disturbances and appetite/eating disturbances. The scripted NPI interview includes a compound screening question for each symptom domain, followed by a list of questions about domain specific behaviors administered when a positive response to a screening question is elicited. Neuropsychiatric symptoms are rated by the caregiver within a domain in terms of both frequency (1=rarely, less than once per week; 2=sometimes, about once per week; 3=often, several times per week; and 4=very often, once or more per day) and severity (1=mild; 2=moderate; 3=severe), thus yielding a composite symptom domain score (frequency × severity) ranging from 0 (absence of behavioral symptoms) to 144 points (maximum severity of behavioral symptoms). Frequency and severity rating scales have anchor points to enhance the reliability of caregiver responses. Caregiver distress is rated for each positive neuropsychiatric symptom domain on a scale anchored by scores from 0 to 5 points (0=no distress; 1=minimal distress; 2=mild distress; 3=moderate distress; 4=severe distress; and 5=very severe distress). The NPI is administered as a structured interview taking roughly 20 minutes.

*Neuropsychiatric Inventory Questionnaire (NPI-Q).* The NPI-Q^14^ is a caregiver-based questionnaire in which the carer indicates the presence or absence of NPS in the patient during the last few weeks. It can be completed in 5 to 10 minutes and assesses the same 12 behavioral symptoms as the NPI; however, unlike the NPI, the NPI-Q only considers the severity of NPS and caregiver distress. The severity scale has scores ranging from 1 to 3 points (1=mild; 2=moderate; and 3=severe) and the scale for assessing caregiver distress has scores ranging from 0 to 5 points (0=no distress; 1=minimal distress; 2=mild distress; 3=moderate distress; 4=severe distress; and 5=extreme distress). The NPI-Q has proven valid and has acceptable levels of internal consistency.^14^

**Other instruments.** In addition, the Addenbrooke's Cognitive Examination - Revised - Chilean Version - (ACE-R-Ch)^20^ and the CDR^18^ were administered as a measure of global cognitive efficiency and dementia severity respectively.

**Procedure and data analysis.** Firstly, the NPI was administered to the primary caregiver of each patient with AD included in this study. Subsequently, the NPI-Q was filled in by the same carer two weeks later. This gap of two weeks between the administration of the NPI and the NPI-Q was due to logistical reasons. It was assumed that this interval had no influence on patients' behavioural changes, as all the participants in this study had stable clinical pictures during the preceding 6 months of assessment.

The statistical analysis was performed with the IBM SPSS 22 package.^21^ Descriptive demographic data and clinical profiles were obtained for the sample. A comparative analysis was conducted to estimate the prevalence of NPS in this sample of AD patients according to the NPI and the NPI-Q. The Chi-squared test was employed to compare and assess whether there were significant differences between the scales in terms of the frequency/percentage of each symptom. The Mann-Whitney U test for independent samples was used to compare the two groups (CDR=1 and 2) and detect significant differences in the means of each NPS.

In order to estimate the convergent validity of the instrument, a correlation analysis was performed according to the distribution of each variable (Pearson's r or Spearman's rho where appropriate) among the NPI (total score, severity, distress, and by domain), the NPI-Q (total score, severity, distress, and by domain), and the ACE-R-Ch. In order to determine the reliability of the NPI-Q, its internal consistency was estimated with Cronbach's alpha.

## RESULTS

**Demographic data and clinical profiles.** Table 1 summarizes the demographic and clinical data of the total sample (n=53). The sample was divided, according to CDR scores, into CDR=1 (n=26) and CDR=2 (n=27). Concerning clinical profiles, significant differences were found between the groups (p<0.05) in global cognitive efficiency measures (ACE-R-Ch), Total NPI, Total NPI-Q, and NPI-Q Distress.

**Table 1:** Clinical and demographic data.

Variable	All (n=53)	CDR=1 (n=26)	CDR=2 (n=27)	Caregivers
Age	73.8±6.87	74.4±6.5	73.3±6.3	63.66±12.66
Education (years)	11.5±4.82	12.2±4.6	10.9±4.9	12.32±5.05
Total ACE-R score*	61.02±16.18	70.2±12.5	52.2±14.4	
Total NPI score*	17.58±20.61	11.6±12.8	23.3±24.9	
NPI - Distress score	8.21±8.63	5.9±7.2	10.4±9.4	
Total NPI-Q score*	8.64±6.54	6.3±4.8	10.5±7.2	
NPI-Q - Distress score*	11.09±9.87	7.4±7.9	14.1±10.4	

Results expressed as Mean±Standard Deviation.

* Groups differ significantly (p> 0.05)

**NPI-Q.** All the patients displayed at least one neuropsychiatric symptom on both the NPI and NPI-Q scales (Table 1).

The correlation between the NPI total score (sum of the products of severity per frequency of all domains) and the NPI-Q total score (sum of the severity scale of all domains) was positive, high, and significant (r=0.73; p<0.01). Similarly, the correlation between the scores on the NPI and NPI-Q distress scales was positive, high, and significant (r=0.715; p<0.01).

Regarding the ACE-R, a negative, moderate and significant correlation index was observed between the ACE-R-Ch and the total NPI-Q score (r=-0.315; p<0.01) (Table 2)

**Table 2:** Inter-scale correlations among the NPI, NPI-Q, and ACE-R-Ch.

Variables	All (n=53)	CDR=1 (n=26)	CDR=2 (n=27)
NPI-Q -Total/NPI - Total^b^	0.730**	0.725**	0.691**
NPI-Q - Distress/NPI - Distress^b^	0.715**	0.671**	0.671**
NPI-Q - Total/NPI-Q - Distress^b^	0.900**	0.818**	0.912**
NPI - Total/NPI - Distress^b^	0.882**	0.834**	0.929**
NPI-Q - Total/ACE-R^a^	-0.315*	n.s.	n.s.
NPI - Total/ACE-R^a^	n.s.	n.s.	n.s.

* p< 0.05;

** p< 0.01;

n.s. Non-significant.

a Pearson's correlation coefficient (r).

b Spearman's correlation coefficient (r).

Correlating the presence of symptoms in terms of the NPI and NPI-Q, the per-domain correlations indicated a range of values between 0.292 and 0.708 (p<0.01). According to the score on each domain, significant correlations were found (p<0.05), ranging from 0.383 to 0.962, for hallucinations, dysphoria/depression, anxiety, apathy/indifference, night-time and appetite/eating disturbances. The distress scale showed the smallest number of symptoms that correlated with each other. Despite this, dysphoria/depression, anxiety, apathy/indifference, and irritability were the only NPS that showed significant values, ranging from p=0.438 to p=0.627 (p<0.05) (Table 3). In addition, the instrument displayed a good level of internal consistency (Cronbach's alpha=0.783).

**Table 3:** Inter-scale correlations between the NPI and the NPI-Q.

Domain	NPI-Q Symptom/NPI	NPI-Q - Total/NPI - Total	NPI-Q - Distress/NPI - Distress
Delusions^a^	0.628**	0.678	n.s.
Hallucinations^a^	0.542**	0.962*	n.s.
Agitation/Aggression^a^	0.413**	n.s.	n.s.
Dysphoria/Depression^b^	0.653**	0.383*	0.438**
Anxiety^b^	0.673**	0.499*	0.594**
Euphoria/Elation^b^	0.292*	--	1
Apathy/Indifference^b^	0.450**	0.433*	0.627**
Disinhibition^b^	0.632**	n.s.	n.s.
Irritability/Lability^b^	0.708**	0.419*	0.496**
Aberrant Motor Behavior^b^	0.522**	n.s.	n.s.
Nighttime Behavioral Disturbances^b^	0.676**	0.861**	n.s.
Appetite/Eating Disturbances^b^	0.580**	0.655*	n.s.

* p< 0.05;

** p< 0.01;

n.s Non-significant.

a Pearson's correlation coefficient (r).

b Spearman's correlation coefficient (r).

**Figure 1:**
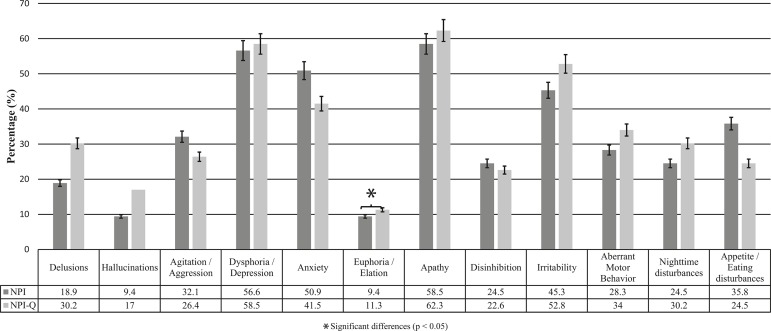
Comparison of the prevalence of neuropsychiatric symptoms in patients with AD using the NPI and the NPI-Q.

The prevalence of the neuropsychiatric symptoms was analyzed using both scales. According to the NPI-Q, the most frequent NPS in AD was apathy/indifference (62.3%), followed by dysphoria/depression (58.5%) and irritability (52.8%). The least frequent NPS in AD were hallucinations (17%) and euphoria/elation (11.3%) (Figure 1).

A significant proportion of the NPS had non-significant differences between the NPI and the NPI-Q. Nevertheless, euphoria/elation was the only NPS whose prevalence differed significantly between the two scales (Chi-squared=4.5; p> 0.05) (Figure 1).

**Figure 2:**
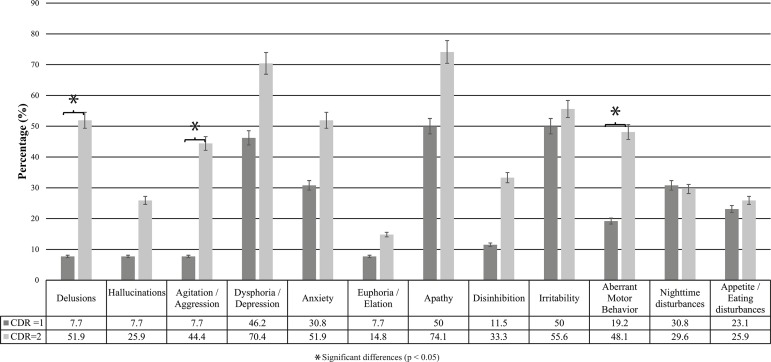
Prevalence of neuropsychiatric symptoms according to the NPI-Q in patients with mild (CDR=1) and moderate (CDR=2) AD.

The comparison of the sample according to the severity of dementia (CDR=1 and CDR=2) revealed an increase in the prevalence of all NPS as the disease progressed, except for night-time disturbances, whose prevalence decreased. The symptoms whose prevalence showed the highest increase included delusions (44.2%), agitation/aggression (36.7%), and aberrant motor behavior (28.9%) (Figure 2). Significant differences were observed between the two groups regarding the presence of delusions, agitation/aggression, and aberrant motor behavior (Figure 2).

## DISCUSSION

The first aim of this study was to examine the psychometric properties and utility of the NPI-Q in a Chilean sample of patients with AD, given that one of the main purposes of this instrument is to facilitate the routine assessment of NPS for dementia cases in everyday clinical practice settings. The second goal of the study was to analyze the frequency of NPS in AD in Chile and to identify any possible differences associated with the severity of dementia.

The following section provides a detailed analysis of these results.

**Psychometric properties.** The NPI-Q showed appropriate convergent validity and internal reliability with respect to total and individual symptom domain scores and caregiver distress ratings on the NPI. Both results are in accordance with those reported in the validation of the original scale^14^ and with those of the Spanish version.^15^

The NPI-Q distress scale was strongly and very significantly associated with the severity of NPS. These results are consistent with those reported in previous studies analyzing the impact of NPS on caregiver burden and quality of life. Kochhann et al.^22^ studied this association and concluded that NPS in patients with AD are the main cause of caregiver distress and burden, and that this association becomes stronger as the severity of dementia increases.

The NPI-Q symptom severity scale showed a strong correlation with both the severity scale and with the composite frequency × severity score (total score) from the NPI, with the correlation index remaining stable regardless of the severity of dementia (once the sample had been divided according to CDR). These results are also in line with the original studies.^14,15^

Thus, the present study provides sufficient evidence to assert that the NPI-Q had acceptable psychometric properties when tested in a cohort of Chilean patients with AD.

**Neuropsychiatric symptom frequency.** According to prior research, over 80% of patients with dementia experience at least one NPS.^6,7^ The results of the present study showed similar indexes, as 100% of the patients included in the sample displayed at least one neuropsychiatric symptom according to the NPI and NPI-Q.

Based on the results obtained in this study, the most common NPS in patients with AD in Chile was apathy/indifference, followed by dysphoria/depression and irritability. In contrast, the least frequent symptoms were hallucinations and euphoria/elation. Only irritability was more prevalent in comparison with the results published by Qing-Fei et al.^23^ These researchers, from a meta-analysis of prior studies, concluded that the most frequent NPS in AD were apathy/indifference, dysphoria/depression, agitation/aggression, anxiety, night-time behavioural disturbances, and irritability, in decreasing order of prevalence.

Our results are consistent with findings of another study conducted in Chile that showed apathy/indifference, anxiety, and irritability to be the most prevalent symptoms in these patients;^24^ in addition, the present study indicated that dysphoria/depression is one of the main NPS observed in AD.

Comparing the prevalence of NPS according to the severity of dementia, our results showed that only night-time disturbances decreased, whereas delusions, agitation/aggression, and aberrant motor behavior increased as the disease progressed. In a longitudinal study, Brodaty et al.^25^ concluded that symptoms such as depression, euphoria/elation, and night-time and eating disturbances did not increase significantly in frequency, but hallucinations, agitation/aggression, aberrant motor behavior, and apathy/indifference, among other symptoms, became more prevalent. By contrast, Bergh and Selbæk,^26^ also in a meta-analysis, concluded that although the course of NPS is heterogeneous, most display some increase, where aggression was the most persistent symptom.^26^

The prevalence of NPS assessed by the two scales differed by 6.1% overall in this study, and depending on the specific domain, tended to be higher or lower on the NPI-Q. This result is consistent with the original validation of the NPI-Q^14^ and its Spanish version,^15^ which showed differences of 5% and 6.7%, respectively. These differences in prevalence between the two scales could be explained by the fluctuations in NPS over time.

The NPI-Q is also useful because it provides an indicator for assessing global and NPS-related caregiver distress; in fact, it has already been used in multiple studies assessing this variable,^27-29^ allowing planning of personalized intervention strategies for caregivers.

**Limitations.** This study was conducted in caregivers/informants with an average educational level where the administration of the questionnaires was aided by professionals when questions were not readily understood. This point is of special consideration for clinical practice, particularly when involving low socioeconomic status population groups whose understanding of the questions may be limited. Likewise, it is advisable for the administration of the NPI-Q to be assisted or alternatively, the instrument can be used as a brief interview, which should facilitate and ensure faithful representation of the patient's reality.

One of the main limitations of our study is the inclusion of only AD patients. Further studies should include other dementia and neuropsychiatric conditions to determine the validity of NPI in other diseases. Nevertheless, previous evidence in other countries supports the extrapolation of the NPI-Q to other forms of dementia.

In summary, given the good psychometric properties and utility of the NPI-Q scale, it can be concluded that the instrument is suitable for assessing NPS in dementias in everyday clinical practice in Chile, having proven being brief, simple, valid, and reliable.
